# Fur removal promotes an earlier expression of involution-related genes in mammary gland of lactating mice

**DOI:** 10.1007/s00360-023-01474-9

**Published:** 2023-01-18

**Authors:** Elżbieta Król, Frances Turner, Davina Derous, Sharon E. Mitchell, Samuel A. M. Martin, Alex Douglas, John R. Speakman

**Affiliations:** 1 School of Biological Sciences, University of Aberdeen, Aberdeen, AB24 2TZ Scotland, UK; 2 Edinburgh Genomics, University of Edinburgh, Edinburgh, EH9 3FL Scotland, UK; 3 Shenzhen Key Laboratory for Metabolic Health, Center for Energy Metabolism and Reproduction, Institute of Biology and Biotechnology, Shenzhen Institutes of Advanced Technology, CAS, Shenzhen, China; 4 CAS Center of Excellence in Animal Evolution and Genetics, Kunming, China; 5 State Key Laboratory of Molecular Developmental Biology, Institute of Genetics and Developmental Biology, CAS, Beijing, 100101 China

**Keywords:** Heat dissipation limit, Milk production, Milk synthesis-related genes, Involution-related genes, Mammary gland involution, Mother-young conflict

## Abstract

Peak lactation occurs when milk production is at its highest. The factors limiting peak lactation performance have been subject of intense debate. Milk production at peak lactation appears limited by the capacity of lactating females to dissipate body heat generated as a by-product of processing food and producing milk. As a result, manipulations that enhance capacity to dissipate body heat (such as fur removal) increase peak milk production. We investigated the potential correlates of shaving-induced increases in peak milk production in laboratory mice. By transcriptomic profiling of the mammary gland, we searched for the mechanisms underlying experimentally increased milk production and its consequences for mother–young conflict over weaning, manifested by advanced or delayed involution of mammary gland. We demonstrated that shaving-induced increases in milk production were paradoxically linked to reduced expression of some milk synthesis-related genes. Moreover, the mammary glands of shaved mice had a gene expression profile indicative of earlier involution relative to unshaved mice. Once provided with enhanced capacity to dissipate body heat, shaved mice were likely to rear their young to independence faster than unshaved mothers.

**Supplementary Information:**

The online version contains supplementary material available at 10.1007/s00360-023-01474-9.

## Introduction

Provisioning young with milk is highly demanding (Clutton-Brock et al. 1989; Speakman 2008). At the tissue level, lactation requires extensive expansion and differentiation of mammary glands, which is initiated during pregnancy (Macias and Hinck 2012; Lee and Kelleher 2016). Milk production is costly in terms of energy, nutrients, bioactive components (immune factors, growth modulators and hormones) as well as water (McClellan et al. 2008; Andreas et al. 2015). Apart from the energy exported in milk, extra energy is also needed to offset milk production inefficiency (Butte and King 2005). Having milk-dependent young may increase the chance of predation due to increased foraging efforts before lactation (capital breeders) or during lactation (income breeders), and by staying in the proximity of young and actively defending young from predators or conspecifics (Holmes 1991; Rӧdel et al. 2008; Larimer et al. 2011; Nunes 2014). Foraging for extensive periods of time may substantially increase the daily costs of locomotor activity and increase thermoregulatory costs by exposing lactating females to cooler or hotter ambient temperatures (Kurta et al. 1989; Tarnaud 2006; Rogers et al. 2021). Together, the high physiological, behavioural, and ecological costs of lactation are at the core of trade-offs between current and future reproduction and contribute to limits on lifetime reproductive success (Gittleman and Thompson 1988; Stearns 1992; Hamel et al. 2010; Festa-Bianchet et al. 2019).

The conflict between the mother and the young over maternal investment during pregnancy and lactation culminates at the time of weaning (Trivers 1974; Rehling and Trillmich 2007; Haig 2010). While weaning allows the mother to exit lactational amenorrhea and prepare for another breeding event (or to focus on concurrent pregnancy), it withdraws resources from the currently reared young, forcing them to become nutritionally independent (Mandalaywala et al. 2014; Hayssen and Orr 2017). Further complication to the process of weaning may be added by within-litter variation in suckling abilities and weight gain in polytocous mammals, likely to spread the weaning over a longer period of time (Paul and Bhadra 2017). Importantly, the cessation of suckling initiates the regression of mammary tissue to the pre-pregnant state necessary for the next breeding event, in a complex process called the post-lactational involution of mammary gland (Strange et al. 1992; Lund et al. 1996; Watson 2006; Macias and Hinck 2012; Watson and Khaled 2020). To experimentally induce and synchronise mammary involution in laboratory mice (*Mus musculus*), all suckling pups need to be removed from non-concurrently pregnant mothers at the same time, typically at peak of lactation (Stein et al. 2004, 2007; Clarkson et al. 2004; Blanchard et al. 2007). Involution is then triggered by the accumulation of milk in the alveolar lumens (milk stasis) and occurs in two distinct phases. The first phase is reversible, lasts ~ 48 h and is characterised by rapid programmed death of mammary secretory epithelial cells with limited alveolar collapse (Green and Streuli 2004; Baxter et al. 2007). If pups are returned to the mother within 48 h of removal, the involution can be reversed and lactation resumes. If pups are not returned, the circulating levels of prolactin decline and involution enters the second phase, which is irreversible, lasts ~ 2 weeks and is marked by extensive mammary tissue remodelling and repopulation by adipocytes (Li et al. 2017; Wang et al. 2018; Zwick et al. 2018). It has been demonstrated that mammary involution may be delayed but not prevented by concurrent pregnancy (Capuco et al. 2002). Any delay not related to concurrent pregnancy or defect in the process of mammary gland involution are likely to perturb the reproductive cycle of the female, potentially affecting her lifetime reproductive success and fitness (Akhtar et al. 2016; Hughes and Watson 2018a; Jena et al. 2019).

Peak lactation is a time during which a female mammal’s milk production is at its highest and is unresponsive to elevated demands from the young (Hammond et al. 1994, 1996; Johnson et al. 2001; Król and Speakman 2003a). The factors limiting the lactation performance have been subject of intense debate due to their implications for understanding many aspects of mammalian evolution (Speakman and Król 2010a, b), human neonatal nutrition (Victora et al. 2016; Huang et al. 2021), and productivity of dairy livestock (Clay et al. 2020). To focus on physiological rather than ecological or behavioural limits to lactation, most studies have been performed in laboratory conditions, with ad libitum food supply, no predation risk and minimal costs of locomotion and thermoregulation. The physiological nature of the limits to lactation during a single breeding event has been recently demonstrated in laboratory mice (Zhao et al. 2020a). Overall, the limit-to-lactation studies aimed to remove the cap on maternal investment at peak lactation by a wide range of manipulations, including changes in (1) total metabolic demand during lactation (adding extra pups, prolonging lactation, making lactating females simultaneously pregnant, and requiring lactating females to run for food), (2) diet quality and composition, (3) environmental conditions (exposure of lactating females to different ambient temperatures), and (4) heat exchange between lactating females and the environment at a fixed ambient temperature (for review and references see Speakman and Król 2005a, 2011; Król and Speakman 2019). Views about the constraints on lactation performance have changed over time. Initially, lactation performance was thought to be limited by the capacity of digestive tract to process the ingested food (Drent and Daan 1980; Peterson et al. 1990; Weiner 1992; Koteja 1996; Sadowska et al. 2019). This was followed by consideration that lactation performance was limited by capacity of the mammary gland to produce milk (Hammond et al. 1994, 1996; Yang et al. 2013; Zhao et al. 2013; Wen et al. 2017). Finally, the concept of a heat dissipation limit (HDL) associated with the capacity of lactating females to get rid of body heat generated as a by-product of processing food and producing milk was developed (Król and Speakman 2003a, b; Król et al. 2003, 2007, 2011; Speakman and Król 2010a, b; Sadowska et al. 2016; Deng et al. 2020; Huang et al. 2020a; Ohrnberger et al. 2020; Zhao et al. 2020b). Lactogenic heat production in laboratory mice is sufficiently high to double their daily energy expenditure at peak lactation (Król and Speakman 2019), leading to the sustainably elevated maternal body temperature (Gamo et al. 2013), a phenomenon reported in several species of lactating rodents and in large domestic animals (Speakman 2008; Hansen 2009). Further increases in heat production that are not balanced by heat loss may put lactating females at risk of developing potentially fatal hyperthermia (Speakman and Król 2010a).

**Fig. 1:**
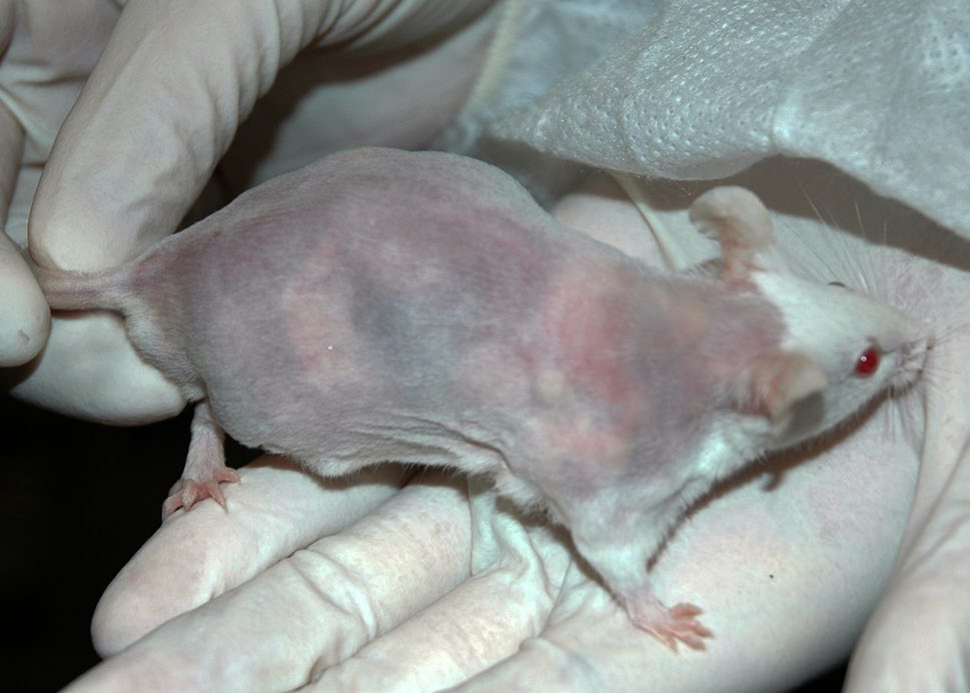
Lactating MF1 mouse with dorsal fur shaved off to increase heat dissipation capacity (photo by John R. Speakman)

An experimental manipulation instrumental for formulating the HDL hypothesis was fur removal to reduce the external insulation of lactating females and thereby elevate their capacity to dissipate body heat (Fig. 1). Shaving off dorsal fur increases the thermal conductance of lactating mice by 10–16% (Zhao and Cao 2009; Sadowska et al. 2019). In MF1 mice that were shaved before peak lactation, food intake increased by on average 12.0% and assimilated energy increased by on average 30.9 kJ day^−1^ compared with unshaved females (Król et al. 2007). With nearly identical mean litter sizes (11.4 pups for shaved and 11.3 pups for unshaved mice), shaved mothers exported on average 15.2% (22.0 kJ day^−1^) more energy as milk than control individuals. The elevated milk production of shaved mice enabled them to wean litters that were on average 15.4% (12.2 g) heavier than young produced by unshaved mice. Since then, shaving-induced increases in milk production have been demonstrated in lactating female bank voles (*Myodes glareolus*) and golden hamsters (*Mesocricetus auratus*) but not in Swiss mice or common voles (*Microtus arvalis*) (Table 1). These contrasting results are consistent with the idea that different species and strains may be constrained by different mechanisms and that the nature of these constraints may depend on the ambient temperature at which the experimental manipulation was performed (Speakman and Król 2011; Huang et al. 2020b).

**Table 1:** Effects of fur removal on lactation performance (maternal food intake, milk production and mass of young) in laboratory and captive rodents

Species/Strain	T_a_^a^ (°C)	Shaving^b^	Food intake^c^	Milk production^d^	Mass of young	Source
Laboratory mouse (*Mus musculus*, MF1)	21	Days 6, 10, 14	↑ 12.0%	↑ 15.2%	↑ 15.4%^e^	1
Laboratory mouse (*Mus musculus*, Swiss)	23	Day 7	↑ 6.9%	Na	↔	2
Laboratory mouse (*Mus musculus*, Swiss)	23	Day 7	↑ 8.8%	↔	↔	3
Common vole (*Microtus arvalis*)	30	Day 2	↔	↔	↑ ~ 5%^f^	4
Bank vole (*Myodes glareolus*)	20	Days 5, 9	↑ 13.2%	↑ 11.8%	↑ 22.1%^g^	5
Yunnan red-backed vole (*Eothenomys miletus*)	25	Day 7	↑ 9.0%	Na	↔	6
Lab mouse (*M. musculus*, Swiss Webster, high BMR)^h^	23	Day 8	↔	Na	↔	7
Lab mouse (*M. musculus*, Swiss Webster, low BMR)^h^	23	Day 8	↔	Na	↓ ~ 40%^f^	7
Golden hamster (*Mesocricetus auratus*)	22	Day 6	↑ 9.9%	↑ 23.4%	↑ 23.7%^i^	8
Laboratory mouse (*Mus musculus*, MF1)	21	Days 6, 10, 14	↑ 18.7%	↑ 19.5%	↑ 19.5%^e^	This study

The difference between shaved (S) and unshaved (U) females is expressed as % of significant increase (↑), % of significant decrease (↓) or no significant change ( ↔) relative to unshaved animals, calculated as (S − U)/U × 100. Data not available are indicated by ‘Na’

Source: 1, Król et al. 2007; 2, Zhao and Cao 2009; 3, Zhao et al. 2010; 4, Simons et al. 2011; 5, Sadowska et al. 2016; 6, Zhu et al. 2016; 7, Sadowska et al. 2019; 8, Ohrnberger et al. 2020

Superscripts and symbols

^a^Ambient temperature during lactation

^b^Day of lactation when shaving was performed (relative to parturition on day 0)

^c^Measured at peak lactation in g/day

^d^Measured at peak lactation in kJ/day

^e^Litter mass at weaning

^f^Pup growth rate

^g^Litter growth rate

^h^Mice selected for high or low basal metabolic rate (BMR)

^i^Pup mass at weaning

∼ Data retrieved from figures

In the current study, we shaved lactating MF1 mice to establish how shaving-induced increases in milk production are mediated at the level of mammary gland transcriptome. We were particularly interested in whether shaving mice to relax the HDL and reduce the risk of maternal hyperthermia affected the time of weaning manifested by involution of mammary gland (mother–offspring conflict). Two aspects of the increases in milk production at peak lactation were also investigated—the milk synthesis machinery at the transcriptomic level, and the mammary gland gene expression correlated with milk production. By RNA-seq profiling of the mammary gland in shaved and unshaved lactating mice, we identified differentially expressed genes (DEGs) associated with shaving and then compared them with sets of genes compiled from the mouse mammary gland literature, containing milk synthesis-related genes and involution-related genes.

## Materials and methods

### Animals and experimental protocol

We used 10 virgin female mice (*Mus musculus* L., outbred MF1) kept on a 12 h:12 h light:dark cycle (lights on 07:00·h) at 21 °C (range 20–22 °C) and a relative humidity of 59% (range 54–64%). Food (CRM, Pelleted Rat and Mouse Breeder and Grower Diet, Special Diets Services, BP Nutrition, Witham, Essex, UK) and water were available ad libitum. At 9–11 weeks of age, mice were acclimated to the single housing environment for 1 week, after which they were paired with MF1 males for 11 days. All females became pregnant and gave birth to young. Following previous convention, the day of birth was counted as day 0 of lactation.

Female body mass and food intake together with litter size and litter mass were recorded every other day from day 4 of lactation to the end of the experiment (day 18). On day 6 of lactation, half of the lactating females (*n* = 5) were shaved, while the other half (*n* = 5) served as an unshaved control group (details below). Milk production was evaluated within the peak of lactation (approximately days 10–18 post-partum, Johnson et al. 2001) from measurements of metabolizable energy intake (MEI) and daily energy expenditure (DEE) by doubly labelled water (DLW) technique (details below). On day 18 of lactation, all mothers were sacrificed by CO_2_ overdose. The right inguinal mammary gland was removed, frozen immediately in liquid N_2_ and stored at − 80 °C prior to RNA extraction. All procedures were authorized by the College of Life Sciences and Medicine Ethics Review Board at the University of Aberdeen and carried out under UK Home Office project licence PPL 60/2881.

### Fur removal

Once the phenotype measurements on day 6 of lactation were completed, all 10 lactating females were anaesthetized with gaseous isoflurane for ~ 10 min. While under anaesthesia, 5 females were shaved dorsally (using a Wella Contura Hair Clipper, Basingstoke, Hants, UK) to remove ~ 72% of fur (Król et al. 2007), as depicted in Fig. 1. Hair regrowth was prevented by repeating the shaving protocol on days 10 and 14 of lactation. The remaining mice were handled and anaesthetised similarly but not shaved.

### Metabolizable energy intake (MEI)

Measurements of MEI were performed on days 12–14 of lactation. Females and their litters were placed in cages with fresh sawdust on day 12 of lactation, and a weighed portion of food was added to the hopper. Samples of the food were taken to determine dry mass content, and the food remaining in the hopper was reweighed on day 14 of lactation. Any uneaten food and faeces were removed from the cage, dried to a constant mass, and weighed. The gross energy content of food and faeces were measured with a Parr 6200 calorimeter using an 1109A semi-micro oxygen bomb (Parr Instrument Company, Moline, IL, USA). MEI was estimated as the difference between energy consumed and defecated, assuming that urinary energy loss was 3% of the digestible energy intake (for details see Król et al. 2007).

### Daily energy expenditure (DEE)

DEE was measured on days 15–17 of lactation, using the DLW technique (Speakman 1997). Lactating females were injected intraperitoneally with ~ 0.25 g of water enriched with ^18^O (28 atom%) and ^2^H (16 atom%). Initial blood samples were taken from the tail tip after 1 h of isotope equilibration to estimate initial isotope enrichments (Król and Speakman 1999); final blood samples were taken 48 h later to estimate isotope elimination rates (Speakman and Racey 1988). Blood samples were immediately heat sealed into glass capillaries and stored at room temperature prior to vacuum distillation. Water from the resulting distillate was used to produce CO_2_ (Speakman et al. 1990) and H_2_ (Speakman and Król 2005b), and the isotope ratios ^18^O:^16^O and ^2^H:^1^H were analysed using gas source isotope ratio mass spectrometry (ISOCHROM μGAS system and IsoPrime IRMS, Micromass, Manchester, UK).

We used the intercept method (Coward and Prentice 1985) to calculate the initial dilution space and the single-pool model (Eq. 7.17 in Speakman 1997) to calculate the rate of CO_2_ production (for details see Król et al. 2007). Energy equivalents of the rate of CO_2_ production were calculated using a conversion factor of 24.026 J mL^−1^ CO_2_, derived from the Weir equation (Weir 1949) for a respiratory quotient of 0.85 (Speakman 1997).

### Milk energy output (MEO)

MEO was calculated as the difference between MEI (days 12–14 post-partum) and DEE (15–17 post-partum) (Król and Speakman 2003b), with MEI being the main determinant of milk production in lactating mice (Speakman 2008). Both MEI and DEE were measured within the peak of lactation (approximately days 10–18 post-partum, Johnson et al. 2001) but on different days to avoid possible changes in behaviour and feeding patterns caused by DLW injection and blood sampling (Speakman and Król 2005b). To reduce possible effects of blood sampling on gene expression, tissue harvest was done on day 18 of lactation (~ 24 h after completion of DLW experiment).

### RNA extraction

Total RNA from the inguinal mammary gland was isolated by homogenization of ~ 100 mg of tissue in TRIzol^®^ Reagent (Ambion by Life Technologies, Carlsbad, CA, USA), using 3 mm tungsten carbide beads and a TissueLyser II Disruption System (Qiagen GmbH, Hilden, Germany). Following isolation, the RNA was quantified by spectrophotometry (NanoDrop Technologies, Wilmington, DE, USA) and its integrity was confirmed by electrophoresis (Agilent Technologies, Santa Clara, CA, USA). All RNA samples had a RIN number > 7.8, meeting the criteria for RNA-seq.

### RNA-seq library preparation and sequencing

RNA-seq library preparation and sequencing were carried at the Beijing Genomics Institute (BGI, Shenzhen, China). The libraries for each of the 10 samples were constructed using the TruSeq Stranded mRNA Sample Preparation Kit (Illumina, San Diego, CA), according to the manufacturer’s instructions. The 50 bp paired-end sequencing was performed on the HiSeq 2000 Sequencing System (Illumina, San Diego, CA) at a sequencing depth of ~ 50 million reads per library. The raw reads were trimmed and converted from BCL to FastQ format with bcl2fastq2 Conversion Software v2.19.1 (Illumina, San Diego, CA). All raw sequences have been deposited in the ArrayExpress repository (http://www.ebi.ac.uk/arrayexpress/) under accession number E-MTAB-11654.

### Read mapping

To assess the quality of the sequencing data, reads were analysed with FastQC v0.11.8 (Andrews 2010). All reads passed the quality control checks and were mapped to the mouse reference genome (GRCm38 release 81) using HISAT2 v2.1.0. (Kim et al. 2015) with the pre-built genome index and default settings for paired-end reads. Alignment rates were above 90%. Aligned reads were counted at gene locations using featureCounts v1.6.4 (Liao et al. 2014).

### Differential analysis of gene expression

Gene expression levels in the mammary glands of shaved and unshaved mice were summarized using principal component analysis (PCA), as implemented by the Bioconductor package PCAtools (Blighe et al. 2018). Differential gene expression analysis was performed using the Bioconductor package edgeR v3.22.5 (Robinson et al. 2010). Both analyses were executed in R v3.5.1 (R Core Team 2018).

To filter out lowly expressed genes, the analyses were performed only on the transcripts with at least 2 counts per million (CPM) in a minimum of 5 samples, amounting to 10,901 such genes in total. Filtered counts were subsequently normalized using a trimmed mean of *M* values (TMM) between each pair of samples. The PCA analysis was performed on the normalized CPM values that have added a prior count = 1 to avoid zeros during calculation of log-values. Scores for individual mice were calculated using the eigenvectors from the first (PC1) and second (PC2) principal components.

The gene expression data (normalized CPM values) were then fitted with a negative binomial generalized log-linear model (glmFit), with the contrast set up to compare shaved vs unshaved lactating mice (*n* = 5 females per group). Differentially expressed genes (DEGs) were identified at false discovery rate (FDR) < 0.05 and absolute Log_2_ FC > 0.5, yielding 752 DEGs in total.

### Overlaps with gene sets from literature

Based on a literature search, we generated 2 sets of genes associated with the transcriptomic changes in the mouse mammary gland during and post lactation, including milk synthesis-related genes, and involution-related genes. The milk synthesis-related gene set was based on the research by Rudolph et al. (2007), Maningat et al. (2009), Mohammad and Haymond (2013), Lemay et al. (2013), Manjarin et al. (2014), Qian and Zhao (2014), Kobayashi et al. (2016), Osorio et al. (2016), Han et al. (2019), Patel et al. (2019), Cayre et al. (2020) and Martin Carli et al. (2020).

The mammary gland involution-related genes were identified by 3 independent microarray experiments (Stein et al. 2004; Clarkson et al. 2004; Blanchard et al. 2007), which were then combined or re-analysed by others (Stein et al. 2007; Bambhroliya et al. 2018). In all 3 studies, the involution of mammary glands was induced by pup removal (Table 2). Because each experiment and reassembly of data had a slightly different protocol and focus of investigation, we generated 4 lists of involution-related genes, according to the outputs from Stein et al. 2004, Clarkson et al. 2004, Stein et al. 2007 and Blanchard et al. 2007.

**Table 2:** Details of the mammary gland involution experiments used to generate involution-related gene sets

Mouse strain	Manipulation	Tissue harvest	Method	Comparison/sample size	Number of involution-related genes	Source
Balb/C	Pup removal (day 7 of lactation)	Days 0, 1, 2, 3, 4, 20 of involution	MA	Days 1, 2, 3, 4, 20 vs day 0 (*n* = 3)	112	1
C57/Bl/6	Pup removal (day 10 of lactation)	Days 0, 0.5, 1, 2, 3, 4 of involution	MA	Days 0.5, 1, 2, 3, 4 vs day 0 (*n* = 3)	130	2
Reanalysis of studies 1 and 2	Reanalysis of studies 1 and 2	Reanalysis of studies 1 and 2	MA	Days 0.5, 1, 2, 3, 4 vs day 0	93	3
CD1	Pup removal (day 12 of lactation)	Days 0, 1 of involution	MA	Day 1 vs day 0 (*n* = 5)	101	4
MF1	Fur removal (days 6, 10, 14 of lactation)	Day 18 of lactation	RNA-seq	Shaved vs unshaved (*n* = 5)	–	This study

The expression of these genes was studied by microarray analysis and refer to the involution of mammary gland induced by pup removal. Details of the shaving experiment (this study) are shown for comparison

Source: 1, Stein et al. 2004; 2, Clarkson et al. 2004; 3, Stein et al. 2007; 4, Blanchard et al. 2007

*MA* microarray gene expression analysis, *RNA-seq* RNA-seq gene expression analysis

Overlaps between DEGs induced by shaving and gene sets from the literature were evaluated using a one-sided Fisher’s exact test. The test was performed on the gene set numbers arranged in 2 × 2 contingency tables, using a function fisher.test in R v3.5.1 (R Core Team 2018), with the parameter ‘alternative’ set to ‘greater’. The *p* values generated by the Fisher’s exact test were subjected to Bonferroni correction for multiple comparisons.

### Functional analysis of gene expression

DEGs in the mammary glands of shaved vs unshaved mice were analysed using Ingenuity Pathway Analysis (IPA, QIAGEN Redwood City, www.qiagen.com/ingenuity). We submitted the whole RNA-seq output (*n* = 10,901 genes, along with their Log_2_ FC and FDR values) to IPA and used this dataset as a reference set for functional analysis of DEGs (*n* = 752, at FDR < 0.05 and absolute Log_2_ FC > 0.5). We used the default analysis settings, apart from species (we selected mice and excluded humans and rats). The focus of the functional analysis of DEGs were (1) canonical pathways, (2) upstream regulators, and (3) downstream effects associated with these genes. The significance of the IPA outputs was based on the Benjamini-Hochberg (B-H) multiple testing correction *p* value, with the overall activation/inhibition states predicted by the IPA z-score algorithm.

### Correlation analysis of gene expression

Counts from mammary gland samples were normalized using the trimmed mean of *M* values (TMM) method with edgeR’s calcNormFactors function. To filter out lowly expressed genes, the correlation analysis was performed only on the genes with at least 5 CPM in at least 2 mice from the shaved group (9279 genes), 2 mice from the control group (9209 genes) and 4 mice from the pooled shaved and control groups (9164 genes). Correlation analysis between mammary gland gene expression (normalized Log_2_ CPM values) and milk production (kJ/day) was performed separately for 5 shaved mice, 5 unshaved mice, and both shaved and unshaved mice (*n* = 10). All analyses were done in R v3.5.1 (R Core Team 2018), using functions cor.test (for 5 mice) and pcor.test (for 10 mice), based on the Pearson’s product moment correlation coefficient. The function pcor.test was used to identify partial correlations between milk production, shaved status, and gene expression. By doing this, the potentially confounding effects of the shaved status were removed (blocked). Correlations between gene expression and milk production were considered significant at FDR < 0.05.

### Statistical analysis of non-transcriptomic data

All non-transcriptomic data were assessed for normality and homogeneity of variance and are presented as mean ± standard deviation (*n* = 5). The whole-body phenotype (body mass, food intake and metabolism) and reproductive performance of shaved vs unshaved mice were compared using Welch two-sample *t* tests. The differences between shaved and unshaved mice in their peak lactation food intake, MEI, DEE, MEO and litter mass were then compared with 95% confidence intervals for the same parameters detected as significant in our original shaving experiment performed on a larger sample size (Król et al. 2007). Measurements repeated on the same individuals (maternal body mass, food intake and litter mass) were analysed using two-way repeated measures ANOVA, with group (shaved and unshaved mice) and day of lactation as factors, and interaction group × day. When the effect of group or interaction was significant, the Holm multiple comparison procedure was applied to determine differences between the groups within each day. Furthermore, the peak lactation performance traits (i.e., food intake, MEI, DEE, MEO and litter mass) were tested for correlation with PC1 and PC2 scores from the PCA analysis of the mammary gene expression. All tests were performed in R v3.6.3 (R Core Team 2018), using default functions (*t* test, anova_test and cor.test).

## Results

### Whole-body phenotype and reproductive performance

Both phenotypic and performance responses to fur removal closely resembled the patterns found in our original shaving study, performed on 20 shaved and 20 unshaved mice (Król et al. 2007). Before shaving, lactating mice that were assigned to shaved and unshaved groups (*n* = 5 females per group) did not differ in their mean body mass or food intake (Table 3, Supplementary Table 1). On average, shaved and unshaved mothers raised a similar number of pups (11.0 ± 1.0 and 11.4 ± 0.5, respectively), with all litter sizes remaining constant from birth to weaning. Likewise, mothers assigned to shaved and unshaved groups did not differ in their litter mass prior to shaving, averaging on day 4 of lactation 34.8 ± 2.3 and 32.2 ± 2.7 g, respectively.

**Table 3:** Phenotypic characteristics and lactation performance of shaved vs unshaved mice (*n* = 5 females per group) before and after fur removal

Parameter	Mean ± SD		Difference
	Shaved	Unshaved	
Before fur removal (first week of lactation)			
Body mass (day 4, g)	45.7 ± 3.0	45.7 ± 3.2	0.0 (0.0%)
Food intake (days 4–6, g/day)	22.0 ± 3.4	20.7 ± 1.6	1.4 (6.6%)
Litter size (day 4)	11.0 ± 1.0	11.4 ± 0.5	− 0.4 (− 3.5%)
Litter mass (day 4, g)	34.8 ± 2.3	32.2 ± 2.7	2.5 (7.8%)
After fur removal (peak of lactation)			
Body mass (day 12, g)	49.2 ± 3.4	48.0 ± 2.2	1.3 (2.6%)
Food intake (days 12–14, g/day)	26.0 ± 4.5	21.9 ± 3.9	4.1 (18.7%)
MEI (days 12–14, kJ/day)	289.5 ± 42.8	250.3 ± 37.2	39.1 (15.6%)
DEE (days 15–17, kJ/day)	123.4 ± 16.1	111.3 ± 11.3	12.0 (10.8%)
MEO (days 12–17, kJ/day)	166.1 ± 41.9	139.0 ± 36.3	27.1 (19.5%)
Litter size (day 16)	11.0 ± 1.0	11.4 ± 0.5	− 0.4 (− 3.5%)
Litter mass (day 16, g)	75.7 ± 7.1	63.3 ± 11.4	12.3 (19.5%)

All parameters were measured during lactation, on days counted from parturition (day 0). The difference between shaved (S) and unshaved (U) mice is calculated as S − U (first value) and then expressed as % of U ((S − U)/U × 100) in the bracket. For statistical details, see Supplementary Table 1

*DEE* daily energy expenditure, *MEI* metabolizable energy intake, *MEO* milk energy output, *SD* standard deviation

Once shaved, lactating mice increased their peak lactation food intake and MEI (days 12–14 post-partum) along with DEE (days 15–17 post-partum) by on average 18.7, 15.6 and 10.8%, respectively, compared with unshaved mothers. As expected, shaved mothers produced on average more milk (by 19.5%) and weaned heavier litters (by 19.5%) than control mice. Despite the same direction and similar magnitude of change as in our original study (Król et al. 2007) (Table 1), the shaving effects in the current study did not reach significance, apart from the litter mass, a proxy for milk production (for details see Supplementary Table 1). Importantly, the differences between 5 shaved and 5 unshaved mice fell within the 95% confidence intervals for the same parameters detected as significant in our previous study with *n* = 20 mice per group.

Two-way repeated measures ANOVA demonstrated that the effects of shaving on litter mass depended on the day of lactation (group, *F*_1,8_ = 6.0, *p* = 0.040; day, *F*_7,56_ = 120.3, *p* < 0.001; interaction group × day, *F*_7,56_ = 2.9, *p* = 0.012). For days 4 and 6 (before fur removal) along with day 18 of lactation, there was no significant difference between the litter mass of shaved and unshaved mice (*p* > 0.05). On days 8, 10, 12, 14 and 16, litters of shaved mothers were heavier than litters of unshaved mothers, by on average 7.4 g (*p* = 0.024), 11.3 g (*p* = 0.027), 12.3 g (*p* = 0.020), 12.5 g (*p* = 0.027) and 12.3 g (*p* = 0.043), respectively (Supplementary Fig. 1). The effects of shaving on maternal body mass (group, *F*_1,8_ = 0.5, *p* = 0.496; day, *F*_9,72_ = 9.1, *p* < 0.001; interaction group × day, *F*_9,72_ = 1.9, *p* = 0.064) and food intake (group, *F*_1,8_ = 2.6, *p* = 0.149; day, *F*_6,48_ = 6.0, *p* < 0.001; interaction group × day, *F*_6,48_ = 1.6, *p* = 0.161) were not significant.

### Mammary gland gene expression

**Fig. 2:**
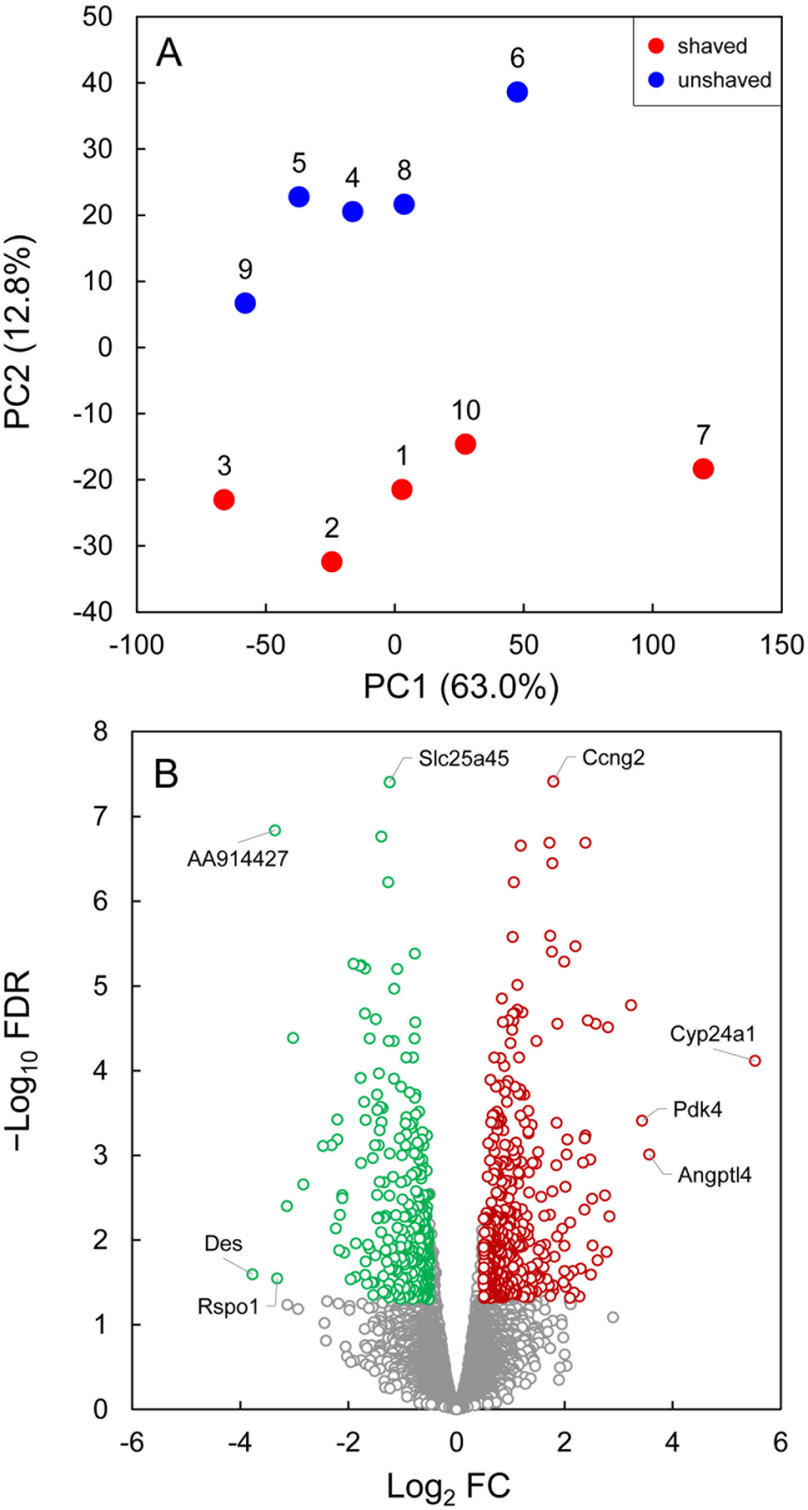
Visualisation of principal component analysis (PCA) and differential gene expression analysis performed on the mammary gland transcriptomes of shaved vs unshaved lactating mice (*n* = 5 females per group). **A** Biplot of the first (PC1) and the second (PC2) principal components, with numbers 1–10 representing the animal ID, and percentage referring to the variance captured by PC1 and PC2 scores. **B** Volcano plot showing 752 DEGs (at FDR < 0.05 and absolute Log_2_ FC > 0.5) that were either upregulated (425 genes in red) or downregulated (327 genes in green) in the mammary glands of shaved mice relative to control mice. The expression of remaining 10,149 genes (in grey) was not significantly different. Gene symbols refer to the most upregulated (Cyp24a1, Angptl4 and Pdk4), downregulated (Des, AA914427 and Rspo1) and significantly altered (Ccng2 and Slc25a45) DEGs (for details see Supplementary Table 3)

PCA results for the mammary transcriptomes of shaved and unshaved lactating mice (*n* = 5 females per group) are summarized in Fig. 2A. The first and second principal components (PC1 and PC2) accounted for 63.0 and 12.8% of the variability in the RNA-seq dataset (matrix of 10,901 transcripts × 10 samples). Both groups of mice showed substantial variability along the PC1 axis, with no clear separation of shaved mice from unshaved controls. In contrast, there was a clear separation between shaved and unshaved mice along the PC2 axis. Neither PC1 nor PC2 scores were significantly correlated with the peak lactation performance traits (Supplementary Table 2).

Differential analysis of gene expression performed on the mammary glands of shaved vs unshaved lactating mice identified 752 DEGs at FDR < 0.05 and absolute Log_2_ FC > 0.5 (Fig. 2B, Table 4, Supplementary Table 3). Among them were 721 protein-coding genes, 19 long noncoding RNA genes and 8 pseudogenes, with other gene types represented by single genes. Out of the 752 DEGs induced by shaving, 425 were upregulated and 327 were downregulated. The Log_2_ FC values associated with these DEGs varied from 5.5 (Cyp24a1) to − 3.8 (Des), reflecting a 46.1-fold upregulation and a 13.7-fold downregulation of gene expression, respectively.

**Table 4:** Results of the differential gene expression analysis performed on the mammary gland transcriptomes in shaved vs unshaved lactating mice (*n* = 5 females per group)

Gene type	Number of differentially expressed genes (DEGs)		
	Upregulated	Downregulated	Total
Protein-coding genes	408	313	721
Long noncoding RNA genes	12	7	19
Pseudogenes	3	5	8
Immunoglobulin gene segments	1	0	1
Antisense long noncoding RNA genes	1	0	1
Unclassified genes	0	1	1
Unmapped genes	0	1	1
All genes (total)	425	327	752

Genes were considered differentially expressed at FDR < 0.05 and absolute Log_2_ FC > 0.5 (for visualisation and details see Fig. 2B and Supplementary Table 3)

### Overlaps between DEGs induced by shaving and gene sets from literature

The literature search for transcriptomic changes in the mouse mammary gland identified 100 milk synthesis-related genes (Supplementary Table 4) and 345 involution-related genes (Supplementary Table 5). The milk synthesis-related genes included prolactin and insulin receptor genes, numerous transcription factors and regulators, as well as transcripts related to the synthesis of the main components of milk such as protein, fat, and lactose (Rudolph et al. 2007; Maningat et al. 2009; Mohammad and Haymond 2013; Lemay et al. 2013; Manjarin et al. 2014; Qian and Zhao 2014; Kobayashi et al. 2016; Osorio et al. 2016; Han et al. 2019; Patel et al. 2019; Cayre et al. 2020; Martin Carli et al. 2020). The involution-related gene set is a compilation of 112 (Stein et al. 2004), 130 (Clarkson et al. 2004), 93 (Stein et al. 2007) and 101 (Blanchard et al. 2007) study-specific genes (Table 2), which amounted to 345 unique involution-related genes.

**Fig. 3:**
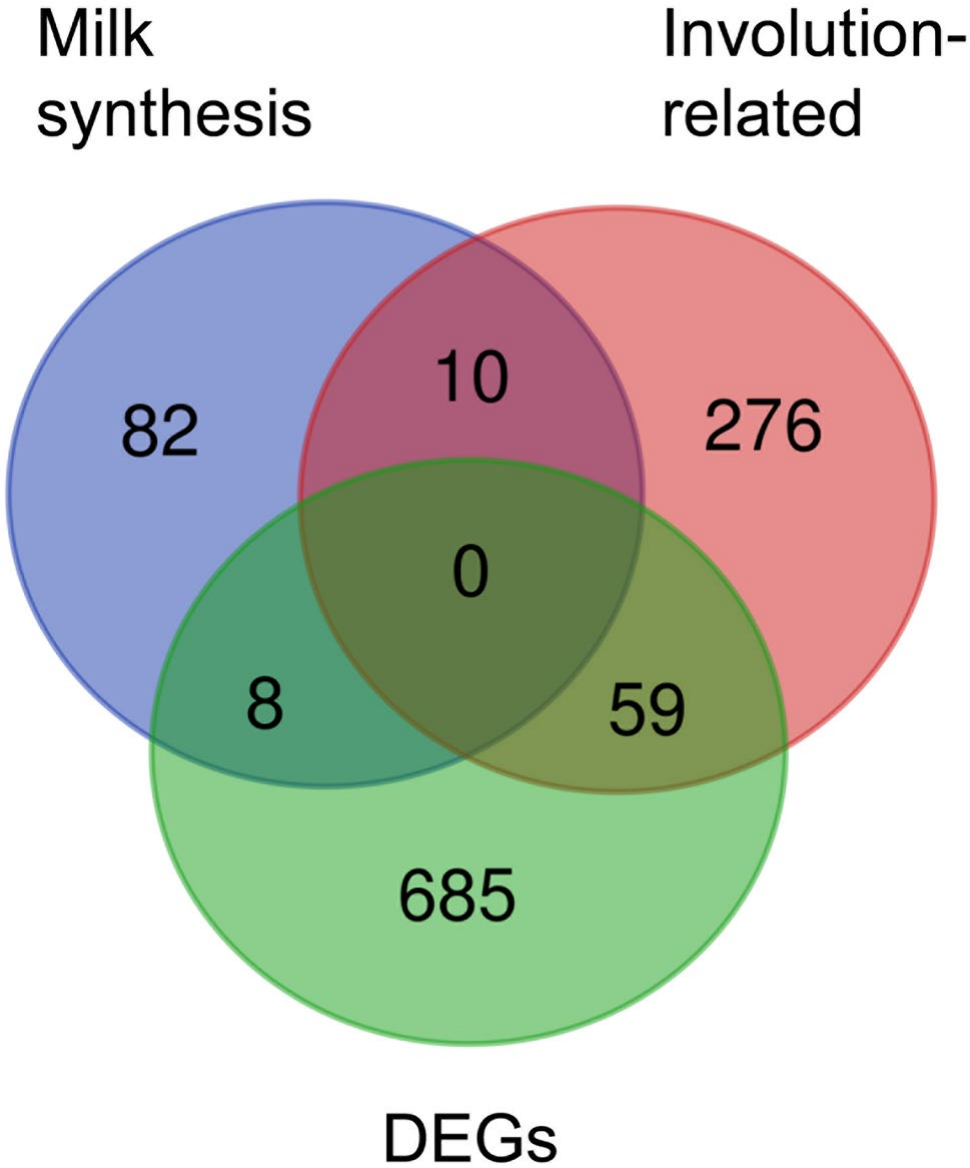
Venn diagram showing the number of common (at intersections) and unique (outside intersections) genes between DEGs in the mammary gland of shaved lactating mice (this study, *n* = 752) and gene sets from literature with milk synthesis-related genes (*n* = 100) and involution-related genes (*n* = 345) (for details see Supplementary Tables 4 and 5)

Comparison of DEGs induced by shaving with the gene sets from the literature revealed 8 and 59 common genes for the milk synthesis-related and involution-related gene sets, respectively (Fig. 3, Table 5, Supplementary Tables 4 and 5). The 8 common milk synthesis-related genes were all downregulated in the mammary glands of shaved mice, but the enrichment of the DEGs with milk synthesis-related genes was not significant (*p* = 0.385) (Supplementary Table 6). In contrast, the overlap between DEGs and the involution-related gene set (59 common genes) was highly significant (*p* = 5.00E − 11), indicating substantial enrichment of DEGs with involution-related transcripts. The majority of the common involution-related genes (52 of 59) were upregulated in the mammary glands of shaved mice, which is consistent with the changes of these genes during involution induced by pup removal, with the magnitude of change (Log_2_ FC values) ranging from 0.5 to 5.5 (Supplementary Table 5). The remaining 7 of 59 common involution-related genes were downregulated in the mammary glands of shaved mice (Log_2_ FC values from − 0.6 to − 2.2).

**Table 5:** Overlaps between gene sets generated in this study and from literature

Gene set (this study)	Milk synthesis-related genes (*n* = 100)	Involution-related genes (*n* = 345)	Shaving-induced DEGs (*n* = 752)
Shaving-induced DEGs (*n* = 752)	8	59*^1^	–
Milk-correlated genes in all 10 mice (*n* = 2)	0	0	0

All gene sets represent mouse mammary gland. The gene sets from this study include DEGs for shaved vs unshaved mice and genes correlated with milk production in both shaved and unshaved mice (*n* = 10) with the fur effect blocked (Supplementary Tables 3 and 10). The literature gene sets include milk synthesis-related and involution-related genes (Supplementary Tables 4 and 5). The overlap is represented by the number of common genes, with the significance evaluated by a one-sided Fisher’s exact test (Supplementary Table 6). An asterisk (*) refers to a significant overlap after applying Bonferroni correction for multiple comparisons (*p* value < 0.05/2)

^1^Significant at *p* value = 5.00E-11

The overlap between DEGs induced by shaving and the involution-related genes was also investigated at the level of study-specific gene lists (Table 2). Comparison of DEGs with the 4 involution-related gene lists revealed 33, 11, 19 and 21 common transcripts for the outputs from Stein et al. 2004, Clarkson et al. 2004, Stein et al. 2007 and Blanchard et al. 2007, respectively (Supplementary Table 5). These overlaps were significant for the gene lists from Stein et al. 2004 (*p* = 3.29E − 13), Stein et al. 2007 (*p* = 1.54E − 05) and Blanchard et al. 2007 (*p* = 4.13E − 06) but not for Clarkson et al. 2004 (*p* = 0.285) (Supplementary Table 7).

### Functional analysis of DEGs induced by shaving

IPA identified 3 canonical pathways that that were significantly altered in the mammary gland of shaved mice at B-H *p* value < 0.05, including p53 Signalling, Docosahexaenoic Acid (DHA) Signalling and IL-23 Signalling (Table 6). These pathways contained 18, 9 and 8 DEGs induced by shaving, which constituted 23.1, 30.0 and 32.0% of all genes that make up p53 Signalling, DHA Signalling and IL-23 Signalling pathways, respectively. Because some DEGs contributed to more than one pathway, the number of unique DEGs present in all three pathways was 23 (17 upregulated and 6 downregulated). None of the pathways were significantly activated or inhibited, with IL-23 Signalling being the closest to the activation state (z-score 1.4).

**Table 6:** Details of canonical pathways altered in the mammary gland of shaved vs unshaved lactating mice (*n* = 5 females per group), identified by Ingenuity Pathway Analysis (IPA)

Canonical pathway	B-H *p* value	Ratio	z-score	Contributing genes^a^
p53 Signalling	0.002	18/78 (0.231)	0.3	Akt1, Akt3, Apaf1, Bax, Bbc3, Ccnd1, Fas, Gadd45a, Gnl3, Hipk2, Pidd1, Pik3c2a, Pik3cb Pik3r1, Sirt1, Thbs1, Tigar, Trp53inp1
Docosahexaenoic Acid (DHA) Signalling	0.032	9/30 (0.300)	–	Akt1, Akt3, Apaf1, Bax, Bik, Cycs, Pik3c2a, Pik3cb, Pik3r1
IL-23 Signalling	0.032	8/25 (0.320)	1.4	Akt1, Akt3, Jak2, Pik3c2a, Pik3cb, Pik3r1, Runx1, Socs3

The analysis was performed on 752 DEGs (for details see Supplementary Table 3). Pathways were considered significantly altered at Benjamini-Hochberg (B-H) multiple testing correction *p* value < 0.05. The ratio is calculated as the number of genes in each pathway that are present in our experimental dataset (contributing genes), divided by the total number of genes that make up that pathway and are present in the reference set. The overall activation/inhibition states of canonical pathways are predicted based on a z-score algorithm; z-score ≥ 2 predicts an increase in the pathway activity while z-score ≤  − 2 predicts a decrease in the pathway activity. No information on the pathway activity is indicated by a hyphen (–)

^a^Gene symbols (in bold) and gene names, followed by Log_2_ FC (in bold)

**Akt1**, thymoma viral proto-oncogene 1, − **0.7**

**Akt3**, thymoma viral proto-oncogene 3, **0.8**

**Apaf1**, apoptotic peptidase activating factor 1, **0.6**

**Bax**, BCL2-associated X protein, **1.2**

**Bbc3**, BCL2 binding component 3, **1.3**

**Bik**, BCL2-interacting killer, **1.2**

**Ccnd1**, cyclin D1, − **1.4**

**Cycs**, cytochrome c, somatic, − **0.6**

**Fas**, Fas (TNF receptor superfamily member 6), **0.8**

**Gadd45a**, growth arrest and DNA-damage-inducible 45 alpha, **2.2**

**Gnl3**, guanine nucleotide binding protein-like 3 (nucleolar), − **0.7**

**Hipk2**, homeodomain interacting protein kinase 2, **0.6**

**Jak2**, Janus kinase 2, **0.6**

**Pidd1**, p53 induced death domain protein 1, − **0.9**

**Pik3c2a**, phosphatidylinositol-4-phosphate 3-kinase catalytic subunit type 2 alpha, **0.8**

**Pik3cb**, phosphatidylinositol-4,5-bisphosphate 3-kinase catalytic subunit beta, **0.6**

**Pik3r1**, phosphoinositide-3-kinase regulatory subunit 1, **0.8**

**Runx1**, runt related transcription factor 1, **1.7**

**Sirt1**, sirtuin 1, **0.9**

**Socs3**, suppressor of cytokine signalling 3, **1.8**

**Thbs1**, thrombospondin 1, **1.9**

**Tigar**, Trp53 induced glycolysis regulatory phosphatase, − **0.7**

**Trp53inp1**, transformation related protein 53 inducible nuclear protein 1, **2.2**

The IPA software predicted 11 upstream regulators responsible for differential gene expression in the mammary glands of shaved mice, at B-H *p* value < 0.05 and absolute z-score ≥ 2 (Table 7, Supplementary Table 8). Among them were 4 transcription factors (Trp53, Cdkn2a, Id3 and Zbtb33) 3 kinases (Jak1, Mapk8 and Egfr), a cytokine (Ifng), an actin-related protein (Actl6a), a peptidase (Zmpste24) and a cytoskeletal linker protein (Gas2l3). Out of the 11 upstream regulators, 7 were activated (z-score ≥ 2) and 4 were inhibited (z-score ≤  − 2). The number of DEGs regulated by each upstream regulator varied from 4 (Zbtb33) to 83 (Trp53). Because some DEGs induced by shaving appeared to be regulated by more than 1 upstream regulator, the number of unique DEGs regulated by all 11 upstream regulators was 144.

**Table 7:** Upstream regulators of gene expression changes in the mammary gland of shaved vs unshaved lactating mice (*n* = 5 females per group), predicted by Ingenuity Pathway Analysis (IPA)

Upstream regulator (gene symbol and name)	Gene type	B-H *p* value	z-score	Target genes^a^
Predicted state of activation (z-score ≥ 2)				
Trp53, transformation related protein 53	Transcription regulator	0.0180	3.4	83
Ifng, interferon gamma	Cytokine	0.0166	2.9	38
Cdkn2a, cyclin dependent kinase inhibitor 2A	Transcription regulator	0.0139	2.2	13
Jak1, Janus kinase 1	Kinase	0.0180	2.2	8
Mapk8, mitogen-activated protein kinase 8	Kinase	0.0044	2.2	13
Id3, inhibitor of DNA binding 3	Transcription regulator	0.0418	2.1	17
Zbtb33, zinc finger and BTB domain containing 33	Transcription regulator	0.0418	2.0	4
Predicted state of inhibition (z-score ≤ − 2)				
Actl6a, actin-like 6A	Actin-related protein	0.0044	− 2.1	8
Zmpste24, zinc metallopeptidase, STE24	Peptidase	0.0431	− 2.2	8
Gas2l3, growth arrest-specific 2 like 3	Cytoskeletal linker protein	0.0316	− 2.4	6
Egfr, epidermal growth factor receptor	Kinase	0.0211	− 2.5	16

The analysis was performed on 752 DEGs (for details see Supplementary Table 3). Upstream regulators were considered significant at Benjamini-Hochberg (B-H) multiple testing correction *p* value < 0.05 and absolute z-score ≥ 2. The z-score ≥ 2 predicts an activation of upstream regulators, while z-score ≤  − 2 predicts their inhibition

^a^Number of differentially expressed genes (DEGs) regulated by each upstream regulator (for details see Supplementary Table 8)

The consequences of transcriptomic changes in the mammary glands of shaved mice were predicted by IPA as 4 downstream effects at B-H *p* value < 0.05 and the number of contributing genes > 100, including Apoptosis, Cell Movement, Solid Tumour and Migration of Cells (Table 8, Supplementary Table 9). The prediction of the activation state was available only for Solid Tumour, with the z-score of − 2.9 indicating a decreased activation state. The number of DEGs contributing to each downstream effect varied from 113 (Migration of Cells) to 150 (Apoptosis), with the total number of unique DEGs associated with all 4 downstream effects being 247.

**Table 8:** Downstream effects predicted from gene expression changes in the mammary gland of shaved vs unshaved lactating mice (*n* = 5 females per group) by Ingenuity Pathway Analysis (IPA)

Downstream effect (biological process or disease)	Functional category	B-H *p* value	Activation state	z-score	Contributing genes^a^
Apoptosis	Cell death and survival	0.012	–	0.0	150
Cell Movement	Cellular movement	0.012	–	1.1	125
Solid Tumour	Cancer	0.012	Decreased	− 2.9	122
Migration of Cells	Cellular movement	0.012	–	0.9	113

The analysis was performed on 752 DEGs (for details see Supplementary Table 3). Downstream effects were considered significant at Benjamini-Hochberg (B-H) multiple testing correction *p* value < 0.05 and the number of contributing genes > 100. The z-score ≥ 2 predicts increased downstream effects, while z-score ≤  − 2 predicts decreased downstream effects; the lack of prediction is indicated by a hyphen (–)

^a^Number of differentially expressed genes (DEGs) that are associated with each downstream effect (for details see Supplementary Table 9)

### Correlation of gene expression and milk production

Correlation analysis identified no genes in the mammary gland of shaved (*n* = 5) or unshaved (*n* = 5) mice that were correlated with milk production at FDR < 0.05 (Supplementary Table 10). When analysis was performed on both shaved and unshaved mice (*n* = 10) with the fur effect blocked, only two genes (Cd276 and Daglb) reached borderline significance at FDR = 0.049. Both these genes were negatively correlated with milk production at correlation coefficients of − 0.976 (Cd276) and − 0.973 (Daglb), and they were not part of milk synthesis-related, involution-related or DEG gene sets (Table 5).

## Discussion

Both humans and other animals are limited in their maximum performance by intrinsic constraints, whether it is growth, reproduction, physical activity or thermoregulation (Drent and Daan 1980; Peterson et al. 1990; Hammond and Diamond 1997; Thurber et al. 2019). Because the physiological limits to performance may also depend on environmental conditions, identifying the mechanisms constraining performance has important ramifications for understanding animal distribution and migration or human athleticism, especially under climate change (Humphries et al. 2002; El Helou et al. 2012; Haïda et al. 2013; Rogers et al. 2021). While many studies were designed to experimentally remove the cap on the performance and then measure the immediate gain in performance (reviewed in Speakman and Król 2005a, 2011; Król and Speakman 2019), the wider context and implications of such gains have not often been investigated. In our previous work, we focussed on the peak lactation performance in MF1 laboratory mice and proposed that lactating females are limited by their capacity to dissipate body heat generated as a by-product of processing food and producing milk (the HDL hypothesis) (Król and Speakman 2003a, b; Król et al. 2003; Speakman and Król 2010a). To remove the performance limit, we shaved off the dorsal fur of the lactating females to enhance their capacity to dissipate body heat and then measured the gain in performance as the shaving-induced increases in food intake, milk production and litter mass (Król et al. 2007). In the current study, we used the same model system to explore the effects of fur removal on the mammary gland, the site of milk production and secretion. By performing RNA-seq profiling of the mammary glands of shaved and unshaved lactating mice, we aimed to understand how the extra milk production is regulated at the level of gene expression.

### Phenotypic responses to shaving

The effects of fur removal on the whole-body phenotype and reproductive performance reported for MF1 mice in the current study were consistent with the results of our original shaving experiment (Król et al. 2007). Furthermore, the shaving-induced increases in food intake, milk production and litter mass in the current study were consistent with the results of shaving experiments performed in lactating bank voles (food intake, milk production and litter mass by 13.2, 11.8 and 22.1%, respectively) and golden hamsters (food intake, milk production and litter mass by 9.9, 23.4 and 23.7%, respectively) (Table 1). Overall, the phenotypic outcome of our shaving experiment was as expected from the previous work (Król et al. 2007; Sadowska et al. 2016; Ohrnberger et al. 2020).

### Transcriptomic responses to shaving

Transcriptome profiling of mammary glands has become a powerful tool for identifying genes and molecular pathways involved in tissue development and function, especially during the cycles of proliferation (pregnancy), functional differentiation (lactation), and death of alveolar epithelium (involution) that occur with each breeding event (Hennighausen and Robinson 2001; Stein et al. 2004, 2007; Clarkson et al. 2004; Blanchard et al. 2007; Cristea and Polyak 2018; Li et al. 2020). Our study demonstrates a link between fur removal and mammary gene expression in lactating mice. Consistent with the other RNA-seq experiments using a small number of biological replicates (Schurch et al. 2016), our study had sufficient power to detect DEGs with larger fold changes (absolute Log_2_ FC > 0.5) but not with smaller fold changes (absolute Log_2_ FC ≤ 0.5) (Fig. 2B). While the majority of RNA-seq analytical tools successfully control their FDR at < 5% for all numbers of replicates, we specifically used edgeR recommended for a lower number of replicates, based on its superior combination of true positive and false positive performances (Robinson et al. 2010; Schurch et al. 2016). As a result, we demonstrated that shaving off dorsal fur in lactating mice significantly altered the mammary expression of 752 genes (Table 4, Supplementary Table 3). For comparison, an exposure of lactating mice to a daily 2 h heat treatment (36 °C) for 14 days was associated with the changes in the mammary expression of 409 genes (*n* = 8 females per group, FDR < 0.01 and absolute Log_2_ FC > 0.6) (Han et al. 2019). In another study, feeding lactating mice with a high-fat diet altered the mammary expression of 628 genes (*n* = 6 females per group, FDR < 0.1 and absolute Log_2_ FC > 1) (Cheng et al. 2018). Using the number of DEGs as a proxy for the magnitude of transcriptomic changes, we conclude that the mammary gland responses to shaving were of similar size to those induced by other whole-animal manipulations, including ambient temperature and diet treatments (Cheng et al. 2018; Han et al. 2019).

### Overlap with milk synthesis-related gene set

The milk synthesis machinery was represented in our study by the milk synthesis-related gene set compiled from the mouse mammary gland literature, containing 4 hormone receptors, 12 transcription factors and regulators, 25 milk protein synthesis-related, 46 milk fat synthesis-related and 13 lactose synthesis-related transcripts (Supplementary Table 4). The 19.5% increase in milk production of shaved mice did not appear to be associated with any substantial changes in the milk synthesis machinery at the level of transcriptome (Table 5, Supplementary Table 6). The lack of significant overlap between mammary DEGs induced by shaving and milk synthesis-related genes may have several explanations. Firstly, there may be other groups of genes involved in the regulation of milk synthesis and its secretion into the alveolar lumen (Ramanathan et al. 2008; Wei et al. 2013). Secondly, a substantial part of such regulation may be post-transcriptional rather than transcriptional (Lemay et al. 2007; Osorio et al. 2016; Mu et al. 2021). Thirdly, milk production of shaved mice may have a different trajectory of changes during lactation than that of unshaved mice. If the latter is the case, then the separation of measurements of milk production and mammary gene expression by a few days may contribute to the data being temporarily mismatched and thus unlinked to each other. In our study, the main determinant of milk production (MEI) was measured on days 12–14 of lactation, while the mammary gene expression was evaluated on day 18 of lactation, assuming no changes in these parameters between approximately days 10–18 post-partum (Johnson et al. 2001).

Of the 100 milk synthesis-related genes, only 8 genes were differently expressed in the mammary gland of shaved mothers, including 1 transcription factor (Srebf1), 5 milk fat synthesis-related genes (Scd1, Fads1, Fads2, Gpd1 and Dhcr7) and 2 lactose synthesis-related genes (Gale and Slc35a2) (Supplementary Table 4). The transcription factor encoded by Srebf1 has been proposed to regulate the expression of genes involved in lipolysis, lipogenesis de novo, fatty acid activation, and triglyceride and cholesterol biosynthesis in the mammary gland during lactation in mice (Rudolph et al. 2010), humans (Mohammad and Haymond 2013) and cows (Ma and Corl 2012), with the confirmed regulation of 3 desaturase genes altered in our dataset (Scd1, Fads1 and Fads2) (Nakamura and Nara 2002). The other milk fat synthesis-related genes with altered expression were involved in glycerol activation (Gpd1) and cholesterol synthesis (Dhcr7), while the changes in the lactose synthesis pathway were associated with the gene regulation of UDP-galactose synthesis (Gale) and transport (Slc35a2).

All these 8 milk synthesis-related genes were paradoxically downregulated in the mammary gland of shaved mice, rather than upregulated as we would have expected in mice producing more milk because of shaving (with Log_2_ FC values from − 0.5 to − 1.2, Supplementary Table 4). Such a decline may indicate a gradual loss of replenishment of the secretory machinery at the mRNA level (Lemay et al. 2007), leading potentially to earlier cessation of milk production and thus earlier completion of the lactation cycle in shaved mice. Our results suggest potentially different trajectories of changes in milk production for shaved and unshaved mice.

### Overlap with involution-related gene set

Using microarray technology, transcriptional profiles of the mouse mammary gland during involution have been studied by numerous research groups (Table 2), providing a basis for the involution-related gene set with 345 transcripts in total (Supplementary Table 5). Comparison of DEGs induced by shaving and the involution-related gene set from the literature revealed a highly significant overlap of 59 protein-coding genes (Fig. 3, Table 5), with the direction of change (52 upregulated and 7 downregulated) closely resembling the expression patterns of these genes in the mice undergoing forced involution following pup removal (Stein et al. 2004, 2007; Clarkson et al. 2004; Blanchard et al. 2007).

At the onset of involution, milk stasis causes distension of the alveolar lumen, which in turn changes the shape of the mammary epithelial cells and increases the local production of leukaemia inhibitory factor (LIF) (Schere-Levy et al. 2003). In shaved mice, LIF gene expression was 6.9-fold higher than in unshaved controls. LIF acts to phosphorylate signal transducer and activator of transcription 3 (STAT3), a master regulator of mammary gland involution coordinating both programmed cell death and removal of dead cells by the immune system (Chapman et al. 1999). Once activated, STAT3 regulates the expression of genes involved in the uptake of milk lipids from the lumen back to the mammary epithelial cells for their degradation in lysosomes, which has been linked to the increased permeability of the lysosomal membranes (Sargeant et al. 2014). This then results in the leakage of cathepsin proteases into the cytosol, activating the lysosomal-mediated programmed cell death (Kreuzaler et al. 2011). Both lysosome-related genes (Scarb2 and Dnase2a) and cathepsin genes (Ctsa and Ctsl) were significantly upregulated in shaved mice. Furthermore, phosphorylation of STAT3 by LIF shifts the balance between pro- and anti-apoptotic signals in favour of programmed cell death, by activation of pro-apoptotic BCL-2 family members and downregulation of PI3K-AKT survival signalling (Hughes and Watson 2018b; Jena et al. 2019). The presence of such shift in the mammary gland of shaved mice was supported by upregulated expression of two pro-apoptotic genes from the BCL-2 family (Bax and Bik) and downregulation of the gene encoding AKT1, a serine/threonine protein kinase involved in regulation of cell survival and proliferation.

The wave of cell death that occurs during the first phase of involution is marked by the increased expression of cell death receptors and ligands (Clarkson et al. 2004; Stein et al. 2007), represented in the mammary gland of shaved mice by a number of significantly upregulated genes, including Fas, Cebpd, Igfbp5, Pik3r1 and Pik3c2a. The gene with the highest upregulation induced by shaving (a fold change of 46.1) was Cyp24a1, associated with the cell death programmes mediated by vitamin D (Lopes et al. 2012). The removal of dead cells from the mammary gland requires the STAT3-mediated switch of mammary epithelial cells from a secretory to a phagocytic phenotype to perform so called non-professional phagocytosis, with the involvement of professional phagocytes such as macrophages (Monks et al. 2008; Akhtar et al. 2016). The presence of phagocytic phenotype of mammary epithelial cells in shaved mice was inferred from the significantly increased expression of genes encoding C/EBPδ (a fold change of 2.2) and CD14 (a fold change of 5.4). In addition, upregulation of Ccl8, Chil1 and Thbs1 genes in the mammary gland of shaved mice was consistent with activation of macrophages (Marion et al. 2016; Urao et al. 2016; Farmaki et al. 2020). The involvement of macrophages in the vasculature remodelling during the regression of mammary gland (Elder et al. 2020) in shaved mothers was also supported by the increased expression of angiopoietin-4 (a fold change of 11.9).

Finally, the permeability of the mammary alveolar tight junctions probably differs between shaved and unshaved mice, as indicated by changes in the gene expression of claudin-1 and claudin-4. Similarly to the involuting mammary gland following pup removal (Stein et al. 2004, 2007; Blanchard et al. 2007), the expression levels of these genes were upregulated in shaved mice. In contrast, we did not detect any shaving-induced changes in the gene expression of matrix metalloproteinases, carboxypeptidases or eosinophils/neutrophil markers, which are part of the transcriptomic signature associated with the second, irreversible phase of mammary gland involution (Clarkson et al. 2004; Stein et al. 2007). Together, our gene expression data strongly suggest that the mammary gland of shaved mothers was already in the process of regressing from secretory to non-secretory phenotype, pointing towards the first (reversible) phase of involution.

### Shaving vs pup removal experiments

Our results raise the question of why the overlap between mammary DEGs induced by shaving (*n* = 752) and the involution-related gene set from the literature (*n* = 345) was not larger than 59 genes, if the mammary gland of shaved mice was indeed involuting. The reasons for such outcome are probably not related to the differences in the statistical power of the contributing experiments as all studies were performed either on 3 mice per time point or 5 mice per group in the single-point experiments (Table 2). Instead, the natural involution accelerated in our study by fur removal and investigated on day 18 of lactation may have a slightly different transcriptomic signature than the forced involution induced by pup removal on days 7–12 of lactation (Stein et al. 2004; Clarkson et al. 2004; Blanchard et al. 2007). The differences between natural and forced involution of mammary gland have been discussed elsewhere (Silanikove 2014). Secondly, even if the transcriptomic profiles of natural and forced involution are similar, forced involution was typically synchronised across the experimental mice (by removing their litters simultaneously), which makes the rapid and dramatic changes in the mammary gene expression easier to detect (Lemay et al. 2007). In contrast, the onset of natural involution is likely to differ in time and intensity between mothers, generating the nonsynchronous expression of the involution-related genes, which in turn increases their inter-individual variability at single time point and thus decreases the chance of detection of these genes as significantly altered. Thirdly, gene expression patterns associated with mammary involution may differ between different strains of mice, as indicated by the significant overlaps between shaving-induced DEGs and the involution-related gene lists from the experiments on Balb/C mice (Stein et al. 2004) and CD1 mice (Blanchard et al. 2007), but not C57/Bl/6 mice (Clarkson et al. 2004) (Supplementary Table 7). Finally, the involution-related gene set from the literature represent the complete process of mammary regression from secretory to non-secretory phenotype (including reversible and irreversible stages of involution), while the DEGs induced by shaving contain only transcripts associated with the early (reversible) involution, which reduces the number of potential genes being in common. Insights into the potential role of shaving-induced DEGs not overlapping with the involution-related gene set (*n* = 752–59) were gained from the functional analysis of gene expression performed on all DEGs.

### Functional analysis of DEGs

Functional analysis of DEGs induced by fur removal identified 3 canonical pathways, 11 upstream regulators and 4 downstream effects that were significantly altered in the mammary gland of shaved mice (Tables 6, 7, 8, Supplementary Tables 8 and 9). The most striking features of our analysis were changes in the p53 tumour suppressor protein in the mammary gland of shaved mice, predicted both at the level of upstream regulators (that drive the observed changes in gene expression) and canonical pathways (that reflect the enrichment of DEGs). Specifically, p53 (encoded in mice by Trp53) was identified as the most activated upstream regulator of DEGs, while p53 Signalling was the most significantly altered pathway. The p53 protein is a master transcription factor that responds to a variety of cellular stresses and regulates key cellular processes such as DNA repair, cell-cycle progression, angiogenesis and apoptosis, with the p53-dependent pathways typically eliminating damaged cells either through apoptosis or cell-cycle arrest (reviewed in Sullivan et al. 2018). The pro-apoptotic role of p53 in mammary gland involution has been demonstrated in a series of mammary-specific knockout studies. The involution programme was delayed in mice with Trp53 null mammary gland by a few days and hyper-delayed (by a few weeks) in mice with Stat3-Trp53 doubly null gland, but successfully executed by p53 in mice with Stat3 null gland, (Jerry et al. 1998, 2002; Chapman et al. 1999; Matthews and Clarke 2005). These studies clearly demonstrate that STAT3 and p53 act together in synergistic manner to assure the regression of the mammary gland and underscore the importance of redundant apoptotic pathways in the involution programme (Allen-Petersen et al. 2010; Yallowitz et al. 2014).

Furthermore, the potential shift from a pro-survival to pro-apoptotic environment in the mammary gland of shaved mice was supported by the predicted activation of upstream regulators such as Ifng (involved in caspase-8-JAK1/2-STAT1-dependent cell death, Woznicki et al. 2021), Cdkn2a (inhibits cell proliferation through LDHA‑mediated AKT/mTOR pathway, Luan et al. 2021), Jak1 (phosphorylates STAT proteins in mammary epithelium, Sakamoto et al. 2016), Mapk8 (promotes expression of involution-related genes, Girnius et al. 2018), Id3 (inhibits cell proliferation and induces apoptosis in vitro*,* Chen et al. 2016) and Zbtb33 (enhances apoptosis in a p53-dependent manner, Koh et al. 2015). At the same time, the predicted inhibition of upstream regulators such Actl6a (an oncogenic driver in many human cancers, Jian et al. 2021) and Egfr (a major regulator of proliferation and differentiation in epithelial cells, Ramírez Moreno and Bulgakova 2022) points towards reduced cell survival and proliferation in the mammary gland of shaved mice.

Apart from p53 Signalling, the other pathways altered in the mammary gland of shaved mice included IL-23 Signalling and Docosahexaenoic Acid (DHA) Signalling, both potentially linked to the increased presence of immune cells in the tissue. IL-23 is a key pro-inflammatory cytokine expressed by activated monocytes, macrophages, dendritic cells and other antigen presenting cells, which signals to activate STAT proteins, predominantly STAT3 (Kortylewski et al. 2009). DHA is suggested to attenuate macrophage death and potentiate efferocytosis, with the net effect of reducing accumulation of cell corpses in the tissue (Rajasinghe et al. 2020).

Finally, Apoptosis, Cell Movement, Solid Tumour and Migration of Cells were identified by IPA as the top downstream effects predicted to be present in the mammary gland of shaved mice. These effects (especially Apoptosis, Cell Movement and Migration of Cells) may reflect processes such as programmed cell death, acute phase response as well as removal of dead cells by professional and non-professional macrophages in the involuting gland (Stein et al. 2004; Pensa et al. 2009). The predicted Solid Tumour downstream effect agrees with the upregulation and activation of tumour-promotional factors in the mammary epithelium and surrounding stroma once lactation ceases (Wallace et al. 2019; Borges et al. 2020). Together, the results of functional analysis performed on gene expression patterns associated with fur removal are consistent with the ongoing involution of the mammary gland in shaved mice.

### Mother-young conflict over weaning

Weaning from lactation is a time when the interests of the mother and the young are likely to differ, with the young expected to benefit from prolonged lactation and larger size while the mother is expected to maximise her fitness by initiating another breeding event (Trivers 1974).

The mechanisms by which prolonged lactation benefits offspring metabolism have been recently uncovered in rats (Félix-Soriano and Stanford 2022; Pena-Leon et al. 2022). In contrast, lactating female rodents typically benefit from having more litters in a short breeding season by being simultaneously pregnant, which sets the duration of lactation to the sufficient minimum rather than to the extended period of time (Roy and Wynne-Edwards 1995). These contrasting interests trigger mother–young conflict because the optimal time for weaning is likely to be later for the young than for the mother, leading to the evolution of complex behaviours such as solicitation displays in the young and the avoidance of offspring by the mother (Kӧlliker and Richner 2001; Fouts et al. 2005; Cox and Hager 2016). Typically, it is the mother who drives the onset of weaning. In house mice (*Mus domesticus*), this starts around day 17 post-partum when the mother starts to rest alone and remains away from the litter (Kӧnig and Markl 1987). Cross-fostering experiments (with natural litters replaced by younger or older pups) demonstrated that the time of weaning may be either fixed relative to the day of parturition (as in guinea pigs, *Cavia aperea* f. *porcellus*, Rehling and Trillmich 2007) or flexible in response to variation in offspring development (as in rats, *Rattus norvegicus*, Nicoll and Meites 1959). However, no experimental studies have investigated the effects of extra milk production on the time of weaning in the mothers with natural litters. Would the mothers with the extra milk production wean the litters earlier than normal to benefit from a shorter interbirth interval or would they rather wean the litters at normal time to benefit from the bigger young? Both the length of interbirth interval and the size of young at weaning are important life history traits that contribute to the mother’s lifetime reproductive success (Clutton-Brock et al. 1989; West and Capellini 2016).

By experimentally increasing milk production in laboratory mice, we demonstrated that on day 18 of lactation, the mammary gland of shaved mice was already involuting, providing strong evidence for shorter lactation and weaning the young earlier than normal, a strategy that could potentially lead to more frequent breeding events in these mice. Based on the chronology of transcriptomic events in the mammary gland following pup removal (Clarkson et al. 2004; Stein et al. 2007), the mammary gland of shaved mice was at the first (reversible) phase of involution that probably started within ~ 48 h prior to tissue sampling. Because the individual pups of shaved mice were on average substantially heavier than the pups of unshaved mice (75.7 g/11.0 pups = 6.9 g and 63.3 g/11.4 pups = 5.6 g, respectively, Table 3), our data are consistent with the idea that the females adjust their reproductive investment according to the size of young (a proxy for quality) by advancing or delaying the time of weaning to reach the minimum size necessary for the young to survive and breed (Kӧnig and Markl 1987; West and Capellini 2016). More work is needed to couple our transcriptomic data with the behavioural manifestations of the weaning, and to establish whether earlier involution of the mammary gland in shaved mice leads to a shorter interbirth interval.

### Study limitations

The results of our study are drawn from a relatively small number of shaved and unshaved mice, with 5 lactating females per group. In consequence, some phenotypic effects associated with fur removal did not reach significance (Supplementary Table 1). The proper interpretation of these results was possible because of the full characterisation of the shaving effects performed in our earlier study, using the four times larger sample size (Król et al. 2007). The transcriptomic results were probably also affected by *n* = 5, mainly by limited statistical power to detect DEGs with relatively small fold changes (Fig. 2B). Yet the pup removal studies with the sample size of 3 mice per time point (Stein et al. 2004; Clarkson et al. 2004) or 5 mice per group in the single-point experiment (Blanchard et al. 2007) were sufficient to characterise the unique transcriptomic signature of the involuting mammary gland. Similarly, our study had sufficient power to recognise that signature, despite different mechanisms behind triggering the regression of mammary gland.

Our experimental design to have a single snapshot of mammary transcriptome to cover both milk production and involution processes did not work as expected. That design was based on our earlier study indicating no changes in MEI and thus milk production between approximately days 10–18 post-partum in MF1 mice (Johnson et al. 2001). In contrast, our current study indicated potentially different trajectories of changes in milk production for shaved and unshaved mice. On day 18 of lactation, the mammary gland of shaved mice was already in the process of regressing from secretory to non-secretory phenotype. As such, changes in the mammary transcriptome on day 18 post-partum no longer represented greater milk production of shaved mice measured on days 12–14 of lactation (Table 5, Supplementary Table 6). A similar mismatch (no correlation) between milk production and mammary gene expression was also observed in unshaved mice as well all mice with the fur effect blocked (Supplementary Table 10), highlighting the need for simultaneous measurements of these parameters in future studies linking transcriptome to function.

## Conclusions

We shaved lactating MF1 mice to increase their milk production (Król et al. 2007) and investigated their mammary gene expression profiles relative to unshaved mice. The focus of the study was to search for transcriptomic clues on the mechanisms underlying the increased milk production and for consequences of the extra milk production for the mother–young conflict over weaning, manifested by advanced or delayed involution of mammary gland. We demonstrated that the mammary glands of shaved and unshaved mice were at different stages of the lactation cycle when sampled. The extensive transcriptomic analysis indicated that the mammary gland of shaved mice had a gene expression profile indicative of earlier involution relative to unshaved mice. Our interpretation of these results is that once provided with the enhanced capacity to dissipate body heat, shaved mice were likely to rear their young to independence faster than unshaved mothers, thereby potentially benefiting from shorter lactation and shorter interbirth interval to maximise their lifetime reproductive success (Clutton-Brock et al. 1989; West and Capellini 2016). Further research is needed to establish the link between earlier regression of the mammary gland and the timing of the next breeding event. We argue that the association between HDL and female fecundity is understudied and should be considered when investigating lactation performance in laboratory and natural conditions.

## Supplementary Information

Below is the link to the electronic supplementary material.

Supplementary Fig. 1 (DOCX 131 KB)

Supplementary Table 1 (XLSX 19 KB)

Supplementary Table 2 (XLSX 22 KB)

Supplementary Table 3 (XLSX 148 KB)

Supplementary Table 4 (XLSX 24 KB)

Supplementary Table 5 (XLSX 474 KB)

Supplementary Table 6 (XLSX 21 KB)

Supplementary Table 7 (XLSX 10 KB)

Supplementary Table 8 (XLSX 22 KB)

Supplementary Table 9 (XLSX 26 KB)

Supplementary Table 10 (XLSX 1589 KB)

## Data Availability

The RNA-seq data generated for this study are available in the ArrayExpress repository (http://www.ebi.ac.uk/arrayexpress/) under accession number E-MTAB-11654. The R script is available from FT on request.
