# Supplementary material for: Fur removal promotes an earlier expression of involution-related genes in mammary gland of lactating mice
Source: J Comp Physiol B. 2023 Jan 18;193(2):171–92. doi: 10.1007/s00360-023-01474-9 (PMC9992052; doi:10.1007/s00360-023-01474-9)
Supplement: Supplementary file 1 — Supplementary Fig. 1 (DOCX 131 KB) [file 360_2023_1474_MOESM1_ESM.docx]

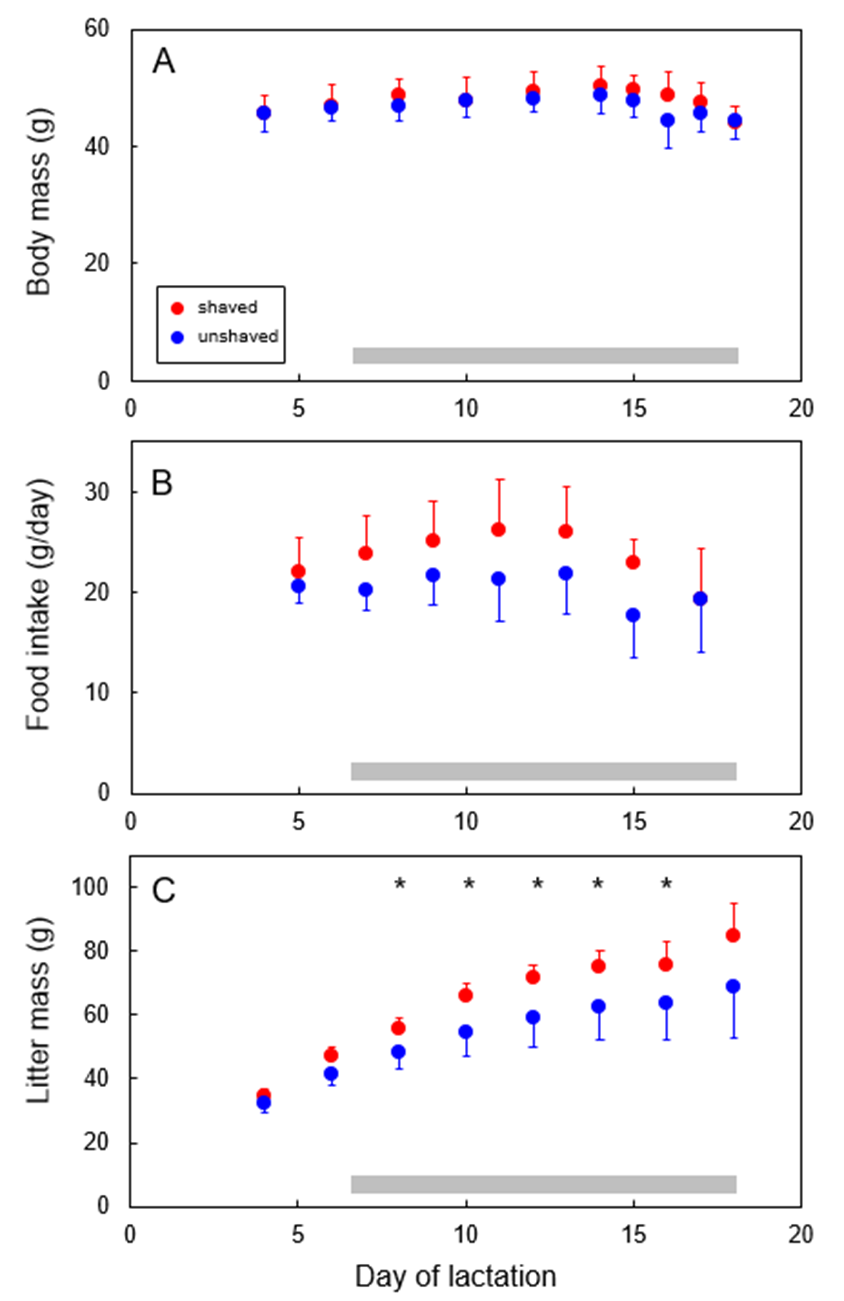


**Supplementary Figure 1.** Body mass **(A)**, food intake **(B)** and litter mass **(C)** of shaved (n = 5) and unshaved (n = 5) mice during lactation. Grey bars indicate period when dorsal fur was removed to enhance capacity to dissipate body heat. Values are means ± SD. Asterisks refer to days of lactation when differences between shaved and unshaved mice were significant (for details see Results).
